# A Novel 3D-Printed Flow Cell Design for *In Operando* Disposable Printed Electrode Replacement: Improving Continuous Methylene Blue Determination

**DOI:** 10.3390/mi17030325

**Published:** 2026-03-05

**Authors:** Željka Boček, Elizabeta Forjan, Andrej Molnar, Marijan-Pere Marković, Domagoj Vrsaljko, Petar Kassal

**Affiliations:** University of Zagreb Faculty of Chemical Engineering and Technology, Trg Marka Marulića 19, 10000 Zagreb, Croatia; zbocek@fkit.unizg.hr (Ž.B.); mmarkovi1@fkit.unizg.hr (M.-P.M.); dvrsal@fkit.unizg.hr (D.V.)

**Keywords:** microfluidics, 3D printing, screen-printed electrode, electrochemical sensor, Methylene Blue, continuous monitoring

## Abstract

Using disposable screen-printed electrodes faces major challenges when attempting to monitor a continuous process, especially in systems where there is pronounced adsorption, fouling, degradation, or in cases of irreversible electrochemical reactions. Methylene Blue (MB) exhibits some therapeutic properties and is commonly used as a redox reporter in DNA sensors, but is also considered a toxic pollutant in aquatic systems. MB demonstrates strong adsorption to carbon materials, which prevents its electroanalytical determination in multiple measurements with a single electrode. Our work details direct electrochemical determination of MB with only the native carbon screen-printed working electrode as sensing material and optimization of the analytical method. In batch mode, we significantly improved sensitivity and interelectrode reproducibility by introducing a prepolarization step, but successive measurements in lower concentrations were not feasible due to strong adsorption. A fully customizable, modular flow cell was 3D printed to allow in operando replacement of the planar screen-printed three-electrode system after measurement during continuous flow. As confirmed by mechanical properties testing, the rigid polyacrylate upper section of the flow cell provides structural stability, combined with a flexible TPU lower section which enables effortless sensor hot swapping and effective sealing during flow. With an optimized hot swapping flow detection method, MB was detected via square wave voltammetry with a sensitivity of 65.59 µA/µM and a calculated LOD of 7.75 nM, which outperforms similar systems from the literature. We envisage this approach can be integrated into low-cost continuous environmental monitoring systems or in-line quality control, especially in flow chemistry synthesis.

## 1. Introduction

Use of additive manufacturing, more commonly known as 3D printing, to fabricate microfluidic devices has been promising, since it allows for rapid prototyping, automated fabrication, incorporation of varying materials and especially integration of fabricated 3D microstructures with other devices, such as microelectronics or sensors, all for a relatively low price tag [1]. While various 3D printing approaches exist, fundamentally differing in the way the precursor material is shaped into the final product, most successfully employed approaches in producing microfluidic devices include inkjet 3D printing (i3DP), fused deposition modelling (FDM), stereolithography (SLA) and two-photon polymerisation (2PP), with each offering unique features for the final printed microfluidic device [2]. From the very beginning, microfluidics has been utilized for applications requiring the use of small sample and/or reagent volumes, with the added benefits of those systems being low cost and allowing operation with little to no special training. Fields benefiting from employing microfluidics range from (bio)chemical analysis, chemical synthesis, point-of-care medical testing and monitoring all the way to food safety and environmental monitoring [3]. Looking past the fabrication angle, 3D printing offers adaptability of the microfluidic system for integration of non-fluidic sensing elements, autonomous components and other functional substrates.

Although researchers have demonstrated the use of solely fluidic microdevices for diverse biochemical applications, the incorporation of microelectronics with microfluidics can greatly extend the capabilities of self-contained lab-on-a-chip technologies [4]. After all, the lab-on-a-chip concept has a goal of integrating sensing devices, such as microelectronics and electrodes, to extend the capabilities of the microfluidic system and allow high throughput and multiplexing [1,4]. Early research printed the microfluidic components as modularized parts which were subsequently assembled with separately prepared electrodes, making most components recyclable for rapid chemical and biological detection [1]. Lab-on-a-chip devices are commonly endowed with sensing capabilities by integrating mass-produced planar, miniaturized and cost-effective electrode systems, such as screen-printed electrodes (SPEs) [5,6]. Printed electrochemical systems are compact and easily integrated into a lab-on-a-chip environment, allowing portability and reducing laboratory dependence, which is especially useful for point-of-care (PoC) detection [7,8]. While in some cases SPEs could be reusable, they are generally considered to be one-shot and disposable, as their low cost of production makes reusability unfeasible compared to swapping one electrode for another [5]. Several strategies for constructing the microfluidic cell have already been employed to allow replacement of the SPE when a new sensor is needed, utilizing magnets [9], nuts and bolts [10] or bobby pins and screws [11] to enable partial or complete disassembly of the cell. However, replacing the used SPE with a new one, regardless of strategy, still necessitates stopping the flow process and partially or fully disassembling the system, wasting time and/or sample fluids and disrupting continuous processes. Moreover, reusability of the SPE is impossible when the electrode surface experiences degradation or fouling (e.g., due to adsorption), or when irreversible sensor modifications are used, such as in the case of Methylene Blue displacement from DNA aptamers [12].

An example of a substance that showcases strong adsorption and is commonly used in irreversible single-shot sensors is Methylene Blue (MB), a potent dye from the phenothiazine family. MB was one of the first antimalarial drugs [13,14] and is used for treatment of methemoglobinemia [13]. Other medical uses in therapeutic doses involve cancer treatment, antimicrobial effects, bactericidal and antiseptic properties. Toxic effects occur at higher doses; these values can be quite low for aquatic and microbial life (no-effect concentration for *Daphnia magna* is evaluated to be 4.7 µg/L). MB is thus considered a wastewater pollutant in areas with a developed textile industry. MB has been known to adsorb strongly to carbon materials, such as activated carbon, carbon nanotubes and graphene [15,16], usually attributed to π-π stacking [14]. Due to its strong adsorptive and light absorbing properties, MB is quite frequently used for photodegradation studies in testing new adsorbents and catalysts [13,16,17,18].

MB can be detected by a wide variety of analytical methods, including capillary electrophoresis, high-performance liquid chromatography, liquid chromatography–tandem mass spectrometry, UV/Vis spectroscopy, electrochemical and surface-enhanced Raman spectroscopy and electroanalytical methods. However, a convenient and effective method to detect low concentrations of MB alone has not been developed so far [19,20].

MB is also widely used for its redox properties. With a reduction potential of ~−0.25 V at pH = 6 vs. Ag/AgCl, it is most commonly used as a redox reporter in DNA-based sensors [21,22], providing a stable electron transfer reaction with a (pH-dependent) reduction potential far from background electrochemical processes [23,24]. However, few works have been dedicated to direct electrochemical detection of MB itself as the analyte, most of which are produced by modifying glassy carbon electrodes (GCEs) with amino-functionalized multiwalled carbon nanotubes (NH_2_-MWCNTs) [25], Ag-MWCNT composite [26], modified metal nanoparticles [20,27] or a composite of both [26]. Other modifications include self-doped TiO_2_ nanotubes [28], silica mesochannels grown on indium tin oxide [19], and various carbon paste electrode fillers and sensing material [17,29,30]. To our knowledge, the only successful example of direct MB detection on printed substrates comes from García-González et al., with detailed characterization of NH_2_-MWCNT functionalized gold SPEs [31].

Our work details direct electrochemical detection of MB with only the native screen-printed carbon working electrode as sensing material, and optimization of the analytical method. However, due to strong adsorption characteristics of MB, particularly on carbon materials, a fully customizable, modular microfluidic flow cell was 3D printed to allow *hot swapping* of the planar screen-printed three-electrode system after measurement, as regeneration or continuous monitoring with one SPE is not feasible. The term hot swapping, taken in from computer science jargon, implies swapping out (adding or removing) components of the system without shutting down the system itself. The same principle is applied here, easily allowing us to replace a disposable printed electrode *in operando*, which enables monitoring of continuous processes with single-shot sensors and facilitates electrode replacement compared to usual microfluidic systems, which do not have an incorporated and mobile sensor ejection system.

## 2. Materials and Methods

### 2.1. Chemicals and Materials

Methylene Blue trihydrate (CAS 7220-79-3, 25 g) was purchased from Thermo Scientific, Kandel, Germany. Potassium dihydrogen phosphate (KH_2_PO_4_) was purchased from Gram-Mol, Zagreb, Croatia. Potassium chloride (KCl) and potassium hydrogen phosphate (K_2_HPO_4_) were purchased from Kemika, Zagreb, Croatia. All chemicals were pro-analysis grade and were used as received. All solutions were prepared using double-distilled deionized water (MilliQ, Millipore, Burlington, MA, USA). Commercial SPEs with a carbon working electrode (ED-S1PE-C10) were purchased from MicruX Technologies, Gijón, Spain. A double junction external Ag/AgCl/3M KCl/3M KCl electrode was obtained from Metrohm, Herisau, Switzerland.

Unless otherwise stated, all solutions were prepared in a phosphate-buffered solution adjusted to pH = 6. This pH value, as well as buffers of different pH (5–8), were prepared by mixing 0.067 M solutions of KH_2_PO_4_ and K_2_HPO_4_ in different ratios to achieve the wanted pH. For measurements demanding a constant chloride background (where the screen-printed Ag was used as a reference electrode after electrooxidation), the solutions were prepared with buffer pH = 6 with 0.1 M KCl. A stock solution of MB was prepared by dissolving a weighted amount of MB powder in a phosphate buffer adjusted to pH = 6, to achieve a stock concentration of 10^−4^ M. The stock solution was diluted accordingly to prepare standard solutions of MB that were used for optimizing measurement parameters and calibrating the electrodes.

### 2.2. Electrochemical Measurements

All electrochemical measurements were conducted on Palmsens4 potentiostat, Palmsens BV, Houten, The Netherlands. Electrochemical method screening was conducted by employing cyclic voltammetry (CV), square wave voltammetry (SWV), differential pulsed voltammetry (DPV) and linear sweep voltammetry (LSV) to measure the signal response on the working carbon SPE. The measurements were first conducted in buffer solution (blank, pH = 6), then in 1 µM MB solution also at pH = 6. CV, DPV and LSV measurements were conducted by varying the scan rate, while SWV was conducted at different square wave frequencies. Method screening showed that the best method was SWV, so all further measurements were conducted with said method with frequency of 600 Hz and amplitude of 25 mV. Repeatability of electrodes used in this work was tested by recording cyclic voltammograms (10 cycles, 50 mV/s) on five different electrodes in 1 µM MB solution also at pH = 6. Unless otherwise stated, all electrochemical measurements were done in a potential range starting from +0.5 V to −0.4 V. For flow measurements, the integrated Ag screen-printed reference electrode was chlorinated via galvanostatic oxidation by applying 2.5 mA current for 10 s in 3 M KCl against external Ag/AgCl/3M KCl/3M KCl reference electrode and platinum wire counter electrode.

Calibration measurements were conducted by recording square wave voltammograms (three consecutive scans in each concentration) in standard MB solutions with the pH value fixed at pH = 6. First, calibration checks were conducted in the concentration range 10^−9^–10^−4^ M to assess the possible linear range and test the system. After initial checks and pretreatment/post-measuring treatment tests, the concentration range was expanded to include more concentrations per decade, starting from 1 × 10^−8^ M. Preconcentration was conducted by applying −0.1 V to the working electrode immersed in the standard MB solution for 120 s. Post-measuring treatment, where performed, was conducted by applying +0.5 V for 600 s to the working electrode in a buffer solution after each calibration curve.

Batch measurements were conducted in a beaker with 20 mL of solution (buffer blank or standard MB solution) with a screen-printed working electrode and external reference and counter electrodes. Flow measurements were conducted by connecting the developed flow system to a Legato210 syringe pump (KD Scientific, Holliston, MA, USA) by loading the syringe with 10 mL of solution and pushing it through the flow system at various flow rates. Interelectrode reproducibility was tested by running a SWV measurement in 1 µM MB solution (pH = 6, 0.1 M KCl), after 1, 2 or 3 min of the solution flowing through the system without preconcentration, or after 2 min of flow with preconcentration at −0.1 V. The measurement was started once the whole volume was replaced with a solution of next higher concentration (~30 s after exchanging the solution at the pump). Each measurement was done in triplicate for three different electrodes. When replacing the electrode with a new one, the flow system was left running and only the mask was ejected to exchange the sensor, preventing unnecessary termination of the flow process and cell disassembly. Real sample measurements were conducted in a tap water sample spiked with a stock solution of the analyte (10^−4^ M), without additional pH or chloride concentration adjustments. Two additions of 25 and 50 µL of stock solution were added to 500 mL of spiked tap water sample to obtain 4 nM and 10 nM addition concentration, respectively.

### 2.3. Flow Cell Design, Fabrication and Characterization

**Figure 1:**
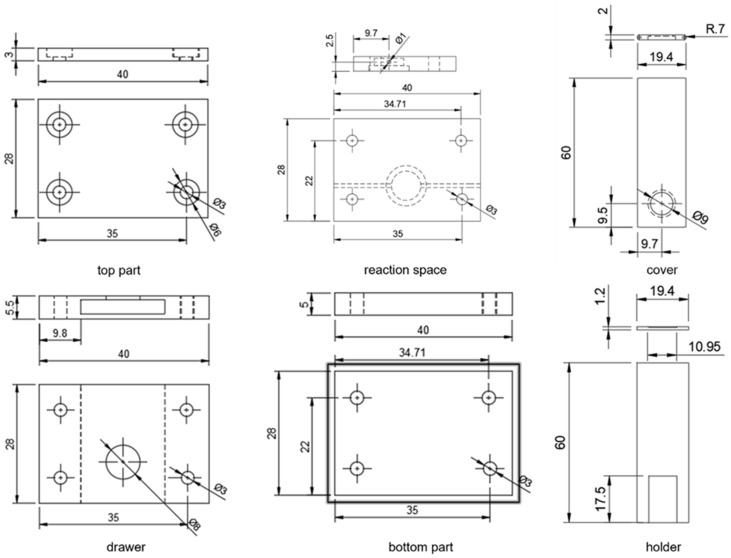
Technical drawings of individual components of the 3D-printed cell, showing dimensions.

Two complementary additive manufacturing processes were used for 3D cell model fabrication. Transparent cell components with microchannels were 3D printed by using digital light processing (DLP), while the flexible components were 3D printed by using fused filament fabrication (FFF). For DLP 3D printing, Anycubic Standard Clear resin (Anycubic, Shenzhen, China) was used as the printing material, and the fabrication was carried out using Anycubic Photon Mono 4 Ultra 3D printer (Anycubic, Shenzhen, China). The printing parameters followed the recommended settings provided by the supplier to ensure accuracy of the printed channel dimensions. The 3D design of the transparent cell components was created in Autodesk Fusion, and the slicing process was performed in Anycubic Photon Workshop, v3.6.0 software (Figure 1). Flexible components were 3D printed using Bambu P1S (Bambu Lab, Shanghai, China) FFF 3D printer equipped with a 0.4 mm nozzle. Thermoplastic polyurethane (TPU) filament (AzureFilm, Sežana, Slovenia) was used, with TPU preset selected in Bambu Studio slicer, v2.4.0.70 software. The nozzle temperature was set to 230 °C, and the heated bed was maintained at 35 °C. Components were printed in a horizontal orientation with 100% infill, using aligned rectilinear pattern to ensure transparency and structural uniformity. Different modules have been prepared to enable easy adjustment of the sample volume according to needs, but only one module is shown in this study.

Both TPU and resin used in the cell fabrication were characterized by Fourier transform infrared spectroscopy using Shimadzu IR-Tracer-100 device (Shimadzu, Kyoto, Japan) equipped with an attenuated total reflection (ATR) cell. The spectra were recorded in transmission mode over the spectral range from 4000 cm^−1^ to 400 cm^−1^ at a resolution of 4 cm^−1^, averaging 20 scans per sample. For the resin samples, post-cured specimens were exposed to an additional UV curing step for 5 min and 15 min after 3D printing (AC cured), whereas under-cured specimens were not subjected to any post-treatment (AC uncured). The mechanical performance of 3D-printed samples was evaluated through tensile testing. All tests were conducted on a Shimadzu AGS-X 50 kN + 500 mm universal testing machine equipped with a long-stroke extensometer (DSES-1000, Shimadzu, Kyoto, Japan). A 5 kN load cell was employed, and the initial grip to grip separation was set to 50 mm. Strain was measured using the DSES-1000 extensometer, with a gauge length of 20 mm. For samples fabricated from Anycubic Standard Clear Resin, a crosshead speed of 50 mm/min was applied, while for TPU samples, a higher crosshead speed of 100 mm/min was used to account for their greater elongation at break.

## 3. Results and Discussions

### 3.1. Electrochemical Characterization of MB on Screen-Printed Electrodes

**Figure 2:**
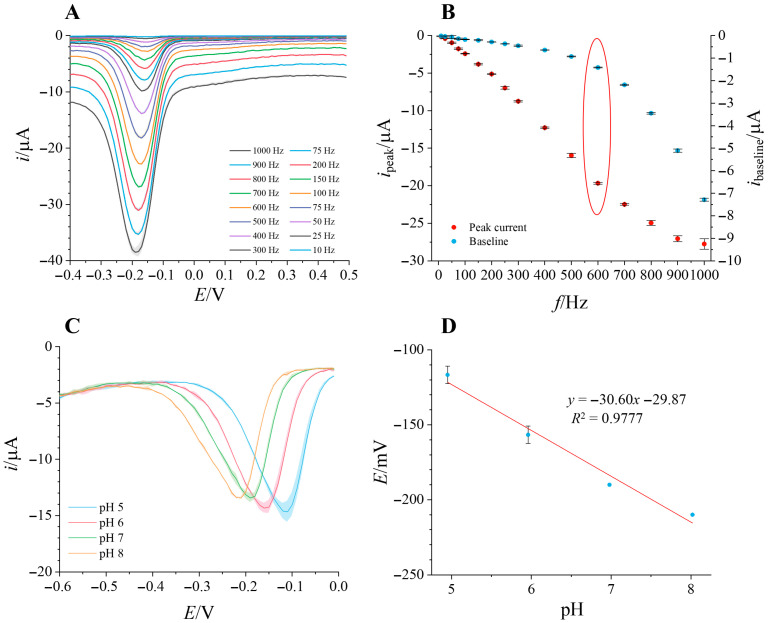
(**A**)—Square wave voltammograms at different frequencies recorded in 1 µM MB, pH = 6; (**B**)—dependence of peak current and baseline potential value on square wave frequency, highlighting the choice of 600 Hz for further measurements; (**C**)—square wave voltammograms recorded in 1 µM MB at varying pH values; (**D**)—dependence of peak potential value on pH.

To assess which electrochemical method is most suited for detection of MB, a screening was performed using four different methods: CV, SWV, DPV and LSV. The observed reaction was the reduction in MB, so the potential was swept from +0.5 V to −0.4 V. Results obtained from CV, DPV and LSV are shown in Figure S1A–C, while the results from SWV are shown in Figure 2. SWV showed the best results compared to the other methods, yielding the highest sensitivity (Figure 2A). A properly defined peak at around −0.15 V, corresponding to MB reduction, was evident at all frequencies tested, with the peak height increasing proportionally to a frequency up to 700 Hz (Figure S1D). However, since the nonfaradaic current also increases with frequency, the final frequency magnitude was chosen by comparing the peak height and baseline potential value, selecting the highest peak current without a significant baseline, taking into account repeatability of the measurements (Figure 2B). Hence, 600 Hz was chosen for all further measurements. Repeatability of different electrodes was tested by recording cyclic voltammetry and comparing the last recorded cycle (Figure S2). For the reduction peak (−29.17 ± 1.50 µA, *n* = 5), RSD is only 5.13%, describing good repeatability of the electrodes used in this work.

Since MB reduction is pH-dependent [24,25], SWV was conducted at different pH values to determine the reaction mechanism (Figure 2C). Dependence of peak potential on the pH value is given by the following equation [25]:

$$\frac{∆E_{p}}{∆pH}=2.303\frac{mRT}{nF},$$

where *m* represents the number of protons and *n* the number of electrons participating in the reduction process. The peak current remains mostly constant with pH, with very slight proportional increase with pH value. The peak potential dependence on pH is shown in Figure 2D, showcasing linear dependence with a slope of −30.60 mV/pH, indicating a one-proton, two-electron reduction mechanism.

### 3.2. Batch Determination of MB

After choosing and optimizing SWV, the working SPE was calibrated in batch mode in the concentration range 10^−9^–10^−4^ M, with external reference and counter electrodes employed at this point. Initially, the lower concentrations of MB either did not give any MB reduction peaks (10^−9^ M), or they showed a current increase without a properly defined peak (10^−8^ M). From concentrations 10^−7^ M and up, the reduction peak becomes fully formed and the peak current height can be properly determined from the baseline. Increasing the signal in lower concentrations has been successfully achieved previously by letting MB accumulate on the electrode [17,31]. However, in our case, even if left to accumulate for a long period of time (up to an hour), the signal in 10^−8^ M MB solution does not increase significantly, with only a slight indication of a peak forming after 30 min in a stationary solution (Figure S3A). We therefore polarized the working electrode at a slightly negative potential to attract the positively charged MB cation, preconcentrating it on the electrode before running a reduction sweep SWV measurement [17,25]. This essentially couples the already sensitive SWV with the adsorptive stripping voltammetry (AdsSV) approach, which is known to improve detection in low concentrations of analytes adsorptive to the electrode surface [32].

Two different potentials (−0.1 V and −0.3 V vs. Ag/AgCl) were applied to the working electrode immersed in 10^−8^ M MB solution (Figure S3B) (more negative potentials did not have any effect). After applying −0.1 V to the electrode for only 60 s, a small peak formed compared to the initial scan. The current magnitude visibly increases when −0.1 V is applied for longer times, while polarizing at −0.3 V either has no effect or even reduces the peak current. Therefore, a potential of −0.1 V was chosen for polarization of the working electrode to preconcentrate MB before the actual measurement. As a compromise between measurement duration and accumulation time, all further preconcentration was conducted by applying −0.1 V for 120 s to the working electrode. However, polarizing the working electrode in even lower concentrations (10^−9^ M MB) did not show any improvement (Figure S3C).

**Figure 3:**
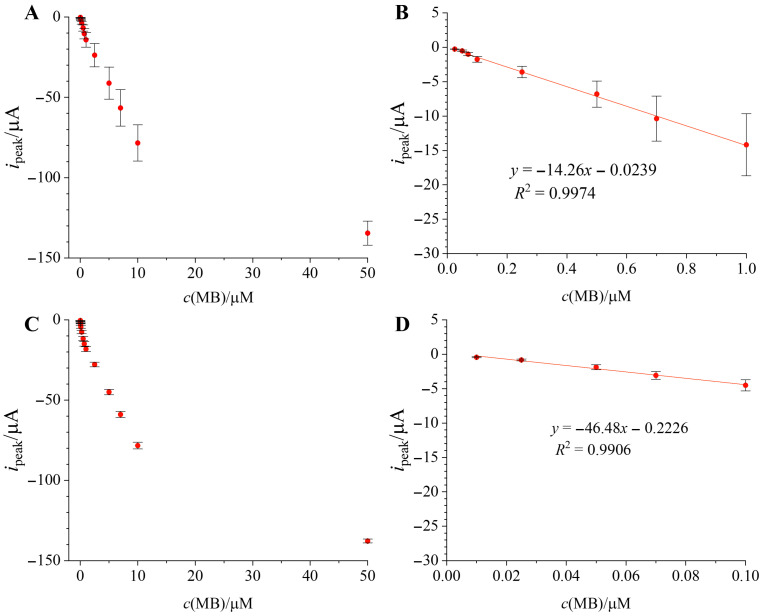
Comparison of batch calibration curves in linear range without (**A**,**B**) and with preconcentration (**C**,**D**), with A and C showing the entirety of the measured calibration curve.

After optimizing preconcentration conditions, a new set of calibration experiments was conducted, comparing results with and without preconcentration. Figure 3A,B show batch calibration results on three separate electrodes without any preconcentration. Hence, error bars are representative of interelectrode reproducibility in both plots in Figure 3. It is evident that the standard deviation between electrodes is large and that the error increases with increasing concentration, indicating poor reproducibility between electrodes. A linear range of 0.025 µM–1 µM was identified when no preconcentration procedure was implemented (Figure 3B), and the electrodes exhibited sensitivity of −14.26 ± 0.32 µA/µM. The limit of detection was determined according to IUPAC guidelines (LOD = 3*σ*/*s*) [33], where *σ* is the standard deviation of the blank (buffer pH = 6) and *s* is the slope of the calibration curve [34] and was found to be 15 nM.

Figure 3C,D show batch calibration results on three separate electrodes with preconcentrating for 120 s at −0.1 V in each concentration. Evidently, preconcentrating MB had a positive effect on detecting lower concentrations (linear range extended towards 0.01 µM), increasing sensitivity (−46.48 ± 2.62 µA/µM) and significantly improving repeatability (greatly reduced standard deviation—for example, RSD reduced from 31.75% to 8.32% in 1 µM MB). The limit of detection has been lowered with preconcentration down to 6 nM. However, preconcentrating also reduced the upper limit of the linear range down to 0.1 µM.

We next evaluated intraelectrode reproducibility by performing three consecutive calibrations with the same electrode. The results are shown in Figure S4. However, it became evident that, despite a washing step, there is a significant adsorbed amount of MB persisting on the electrode surface; in the second and third calibration series, a characteristic peak for MB reduction appears at a much lower concentration than anticipated and observed in the first series. This essentially confirms that consecutive calibration on the same electrode would give inaccurate results in lower concentrations, negatively impacting intraelectrode reproducibility [31].

**Figure 4:**
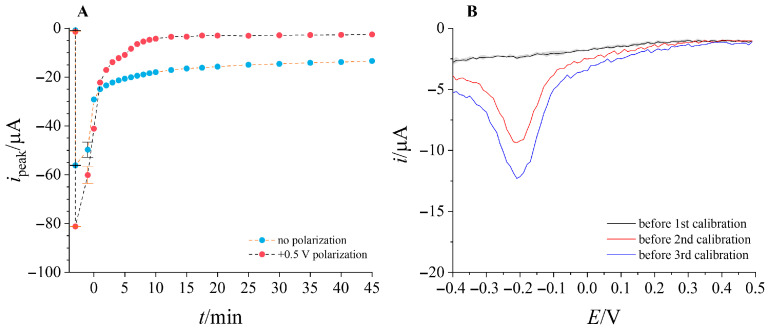
(**A**)—Comparison of electrode signal regeneration with or without polarization; (**B**)—square wave voltammograms recorded in phosphate buffer at pH = 6, before each calibration series shown in Figure S5A.

We therefore tried to recover the electrode surface for subsequent calibrations and an electrochemical washing step was proposed. As MB is present in cationic form, the working electrode was polarized at a positive potential for a set amount of time after. Multiple potential values were tested, with the highest degree of signal recovery obtained at +0.5 V. Figure 4A shows a comparison of MB desorption in time, after an initial experiment of running a SWV measurement in 10^−7^ M, then 10^−4^ M and finally in 10^−7^ M again, tracking the peak current value as an indication of MB desorption. In both cases, the peak current reaches a stable value (−14.45 ± 0.83 µA in case of no polarization, −3.13 ± 0.51 µA in the case of polarization, respectively), but with applied polarization it happens much faster and lower peak current values are obtained, indicating stronger desorption and regeneration of signal. However, even with polarization, the peak current does not fall to the initial value (−1.15 ± 0.40 µA), indicating the presence of a more strongly adsorbed MB layer. This suspicion was confirmed when subsequent calibrations were repeated (results shown in Figure S5). The working electrode was polarized at +0.5 V for 600 s in buffer solution after each calibration series. While the polarization has regenerated the signal in 10^−6^ M, the adsorbed MB persists in contaminating the electrode surface, hindering detection in low concentrations if consecutive calibration is attempted. The effect is shown in greater detail in Figure 4B, showing the increasing reduction peak after each calibration run recorded in buffer solution with no MB, despite post-measuring treatment.

### 3.3. Flow Cell for Sensor Hot Swapping

**Figure 5:**
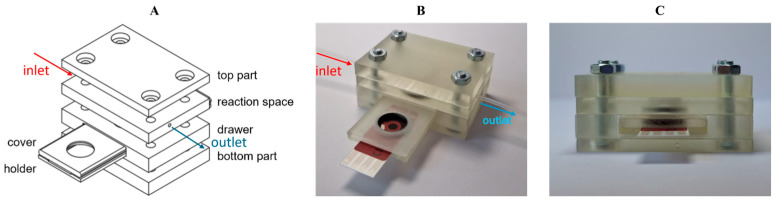
Model of the assembled flow cell. (**A**)—Exploded design; (**B**)—mask ejected, exposing a three-electrode sensing system; (**C**)—mask inserted, side view.

**Figure 6:**
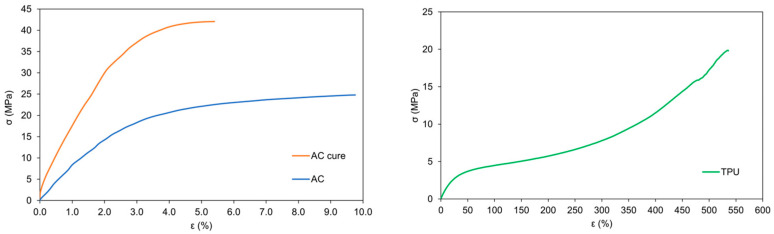
Stress–strain representative curves for Anycubic standard resin (AC), cured Anycubic Standard resin (AC cure), and TPU test samples.

Due to the strong adsorptive properties of MB, a custom flow cell was constructed and 3D printed to circumvent those issues (Figure 5). Since adsorption on the electrode prevents continuous measurements or even successive calibration, which is detrimental to intraelectrode reproducibility, we constructed the cell which allows hot swapping of disposable electrodes. The top and bottom parts, drawer and reaction space were 3D printed using a rigid polyacrylate resin. Channels leading to the reaction space are 1 mm in diameter, and the reaction space itself has a volume of 100.5 mm^3^ (Figure 1), but the design can be exchanged depending on application. The movable parts, consisting of cover and holder, were made of flexible TPU to keep the sensor in place while measuring, but enable easy swapping without leakage while the mask is pulled out. The rigid cover covers the reaction chamber, preventing the fluid from moving out of the reaction space to anywhere but the outlet. Before using it for flow measurement, the flexible TPU bottom holder and rigid upper cover were joined by curing them together with an interphase layer of resin, leaving only a small portion of the flexible TPU layer mobile to enable sensor exchange while preventing the sample from seeping between the two layers. The chemical composition of the polyacrylate resin and TPU was confirmed by Fourier transform infrared spectroscopy (Supplementary Materials) [35,36]. The mechanical properties of the materials used for fabricating the 3D-printed cell were evaluated to ensure sufficient rigidity and flexibility. The upper part of the cell, printed from rigid polyacrylate resin (AC and AC cure), provides structural stability and sealing integrity, while the lower part, made from TPU, offers flexibility and mechanical resilience during sensor replacement and operation. The results from mechanical testing are summarized in Table 1 and Figure 6.

The mechanical characterization revealed distinct differences between the polyacrylate resin (AC and AC cure) and the TPU samples. The uncured polyacrylate (AC) exhibited a moderate elastic modulus (*E*) of 589 ± 77 MPa and a maximum stress (*σ*_M_) of 24.8 ± 2.0 MPa, with a corresponding strain at maximum stress (*ε*_M_) of 9.76 ± 4.35%. The stress at break (*σ*_B_) and strain at break (*ε*_B_) were similar, indicating limited ductility and relatively brittle character. Upon curing, the polyacrylate resin (AC cure) became significantly stiffer and stronger, as reflected by the increase in *E* to 1208 ± 69 MPa and *σ*_M_ to 42.1 ± 1.2 MPa, while *ε*_B_ decreased to 5.4 ± 1.7%. This shift demonstrates the expected transition toward a more cross-linked, rigid structure. Despite the higher stiffness and strength, the energy absorbed prior to fracture (*W*) remained nearly constant (0.37 ± 0.13 J), suggesting limited improvement in overall toughness. In contrast, the TPU exhibited a markedly lower *E* of 12 ± 2 MPa, confirming its high elasticity, while maintaining a comparable *σ*_M_ of 19.9 ± 1.3 MPa. Its strain at break (*ε*_B_) exceeded 500%, and the energy absorption capacity (*W*) reached 10.81 ± 2.12 J, over 10 times greater than that of polyacrylate materials. This pronounced ductility and energy absorption demonstrate the material’s ability to withstand large deformations and recover without damage, validating its suitability for flexible components. The combination of the rigid, high-strength polyacrylate resin and soft, resilient TPU provides a well-balanced mechanical design for the 3D-printed cell. The upper rigid section ensures dimensional stability and effective sealing during operation, while the lower flexible section allows reliable sensor retention and effortless replacement. Hot swapping demonstration is available as Supplementary Video S1. The use of 3D printing thus enables the fabrication of fully customizable cell architecture that integrates mechanical robustness with adaptability, essential for reproducible measurements and long-term device durability.

### 3.4. Flow Determination of MB with Hot Swapping

**Figure 7:**
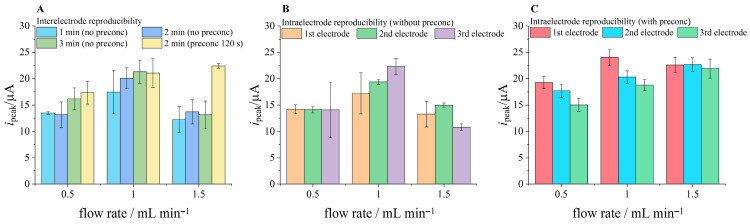
Hot swapping reproducibility at different flow rates and accumulation times. (**A**)—Interelectrode reproducibility after 1, 2 or 3 min of accumulation without preconcentration and 2 min of preconcentration at −0.1 V; (**B**)—intraelectrode reproducibility without preconcentration at different flow rates; (**C**)—intraelectrode reproducibility with preconcentration at different flow rates.

Interelectrode reproducibility is crucial for the flow system proposed in Section 3.3. to work properly. Hence, hot swapping was directly tested in a thorough experiment exploring different accumulation times (1–3 min without preconcentration), flow rates (0.5, 1 and 1.5 mL/min) and preconcentration approach (2 min at −0.1 V) also at different flow rates. The screen-printed Ag electrode was previously coated with AgCl via electrooxidation described in the Experimental Section. All hot swapping was done *in operando*, without interrupting the flow (the syringes were refilled when changing the flow rate; the flow was not stopped while exchanging either electrode). Figure 7A shows interelectrode reproducibility at different flow rates after 1–3 min of accumulation or after 2 min of preconcentration. With the highest peak current value and lowest deviation (1.87%), a flow rate of 1.5 mL/min with 2 min of preconcentration at −0.1 V was ultimately chosen for final flow calibration experiments. Figure 7B shows intraelectrode reproducibility (three consecutive measurements) at each flow rate. The error bars represent the standard deviation of all three measurements (after 1, 2 and 3 min of flow) on one electrode. Three electrodes were used per flow rate to ensure all possible repeatability was tested. Similarly, Figure 7C shows intraelectrode reproducibility of three consecutive measurements after 2 min of preconcentrating at −0.1 V. While increasing the flow rate has a positive effect on peak current and a negative effect on interelectrode reproducibility when no preconcentration is applied, increasing the flow rate when preconcentrating seems to have a positive effect on inter- and intraelectrode reproducibility.

**Figure 8:**
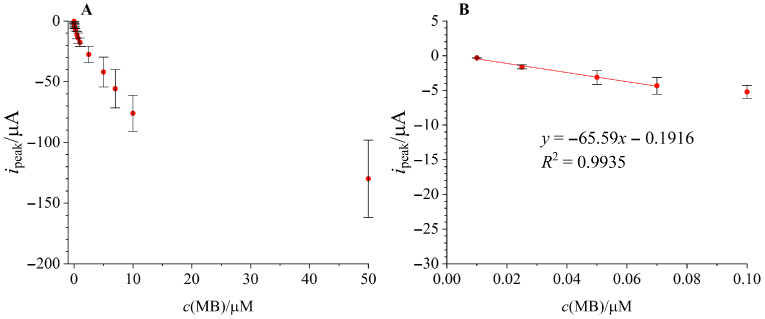
Flow calibration curves obtained in flow mode at 1.5 mL/min on three separate SPEs, with preconcentration for 120 s at −0.1 V. (**A**)—Full calibration curve in the entire tested concentration range; (**B**)—calibration curve in the linear range.

Finally, the flow system was put to the test by calibrating three separate electrodes by pumping the standard solutions through the system with a syringe pump. Preconcentration was applied in all concentrations for 2 min at −0.1 V. Figure 8A shows the entire calibration curve, while Figure 8B shows the linear range with error bars representing interelectrode reproducibility. The linear range (0.01–0.07 µM) is slightly narrower than in batch measurements, but employing the flow system resulted in higher sensitivity (−65.59 ± 3.76 µA/µM) compared to batch mode calibration. The LOD was calculated to be 7.75 nM. To compare the performance of our sensing system with other sensors available in the literature, a short literature data review highlighting the electrode material, linear range, sensitivity and LOD is listed in Table 2. It is evident that, when compared to systems that are modified and hence require more complex assembly, our system using only a commercial carbon SPE without additional enhancing components has achieved superior sensitivity with a low LOD value in a comparable linear range.

To demonstrate practical applicability of the flow cell, a standard addition method was used for a spiked tap water sample. No additional adjustment of pH, ionic strength or chloride concentration was conducted. Two known additions were added to the spiked tap water sample, and together with the original spiked sample the flow measurements were conducted as for the calibration in Figure 8. The results of the standard addition method procedure are shown in Figure S6, and the calculated recovery for the sample is 102.23%, demonstrating satisfactory results for real samples without any additional processing or adjustments.

## 4. Conclusions

Electrochemical detection of MB was thoroughly investigated on commercial carbon SPEs in batch mode. A pretreatment comprising polarizing the working electrode at −0.1 V for 120 s was utilized to locally preconcentrate MB on the working electrode, successfully enhancing the reduction signal, lowering the LOD and significantly improving interelectrode reproducibility. Nevertheless, significant adsorption of MB on the electrode surface was observed, which prevented multiple measurements with the same electrode. A flow cell which enables hot swapping of the SPE without interrupting the flow process was designed and 3D printed. The successful implementation of the flow cell for MB detection is underpinned by the careful selection of the 3D-printed materials. The rigid polyacrylate upper section provides structural stability and effective sealing, ensuring leak-free operation under flow, while the flexible TPU lower section enables effortless sensor insertion and hot swapping without compromising electrode integrity.

Inter- and intra-electrode reproducibility were thoroughly tested by hot swapping electrodes at different flow rates, with and without preconcentration. Optimal results, meaning the highest signal with the lowest error (1.87% interelectrode RSD) were obtained for SPEs undergoing 120 s pretreatment at −0.1 V at a flow rate of 1.5 mL/min. Optimized flow measurements yielded a linear response in the concentration range of 0.01–0.07 µM, with sensitivity improving even more compared to batch calibrations (−65.59 ± 3.76 µA/µM). In such a manner, superior sensitivity and comparable LOD values were obtained in a very low concentration range, utilizing only the carbon SPE without additional modification. Furthermore, our flow system offers reproducible sensor hot swapping, enabling tracking processes that are not convenient for continuous monitoring. Therefore, this system could be successfully utilized for tracking processes without system disassembly, using only small amounts of sample for discrete measurements, which reduces waste and losses and could be beneficial during environmental monitoring or flow synthesis of expensive reagents.

## Figures and Tables

**Table 1:** Mechanical properties of materials used in 3D printing.

Material	*E* (MPa)	*σ*_M_ (MPa)	*ε*_M_ (%)	*σ*_B_ (MPa)	*ε*_B_ (%)	*W* (J)
AC	589 ± 77	24.8 ± 2.0	9.76 ± 4.35	24.8 ± 1.3	9.76 ± 5.23	0.39 ± 0.19
AC cure	1208 ± 69	42.1 ± 1.2	5.40 ± 0.67	42.1 ± 1.0	5.40 ± 1.67	0.37 ± 0.13
TPU	12 ± 3	19.9 ± 1.3	535.31 ± 5.32	19.5 ± 2.4	535.31 ± 19.15	10.81 ± 2.12

**Table 2:** An overview of electrochemical Methylene Blue sensors available in the literature.

Electrode	Sensing Material	Reaction	Method	LR/µM	Sensitivity/µA µM^−1^	LOD/nM	Ref.
ITO	Vertically ordered silica mesochannels	Reduction	DPV	0.01–1	−12.179	4.1	[19]
CPE	Thiol-functionalized clay	Oxidation	CV	1.0–14	N/A	400	[17]
Titanium	Self-doped TiO_2_ nanotubes	Reduction	CV	1.0–94.4	−1.70	900	[28]
GCE	TiO_2_	Oxidation	DPV	0.01–10	0.407	3	[37]
GCE	Nafion stabilized ibuAuNp *	Oxidation	DPV	0.01–1.1	21.227	3.9	[20]
GCE	Calix[4]resorcinarene capped AgNP	Oxidation	DPV	0.001–0.03	24.118	0.16	[27]
GCE	NH_2_-MWCNT	Oxidation	SWV	0.01–0.5	10.5	0.21	[25]
Au SPE	NH_2_-MWCNT	Reduction	SWV	0.1–10	−5.029	100	[31]
Au SPE	NH_2_-MWCNT	Reduction	DPV	0.1–50	−1.388	100	[31]
Carbon SPE	Carbon SPE	Reduction	SWV	0.01–0.07	−65.59	7.75	This work

* Ibuprofen gold nanoparticles.

## Data Availability

Data will be made available from the authors upon request.

## Supplementary Materials

The following supporting information can be downloaded at: https://www.mdpi.com/article/10.3390/mi17030325/s1, Figure S1: Electrochemical screening results for cyclic voltammetry (A), differential pulsed voltammetry (B) and linear sweep voltammetry (C); Δ*i*_peak_ for each tested frequency using square wave voltammetry (D); Figure S2: Repeatability of cyclic voltammograms for five different electrodes; Figure S3: A—Effect of accumulation time in 10^−8^ M MB solution with no additional polarization, B—Effect of different preconcentration times and potentials applied to the working electrode immersed in 10^−8^ M MB solution, C—Effect of preconcentration at −0.1 V applied to the working electrode immersed in 10^−9^ M MB solution; Figure S4: Comparison of three consecutive calibrations conducted with square wave voltammetry without additional treatment (f = 600 Hz, pH = 6); Figure S5: Comparison of three consecutive calibrations conducted with square wave voltammetry with positive polarization between calibrations (f = 600 Hz, pH = 6); Figure S6: Standard addition method plot; Figure S7: FTIR spectra of Anycubic Standard resin and TPU; Figure S8: FTIR spectrum of cured Anycubic resin; Video S1: *in operando* hot swapping demonstration.

## Abbreviations

The following abbreviations are used in this manuscript:

2PP	two-photon polymerisation
AC	Anycubic standard resin
AdsSV	adsorptive stripping voltammetry
Ag-MWCNT	silver-multiwalled carbon nanotubes composite
CV	cyclic voltammetry
DLP	digital light processing
DNA	deoxyribonucleic acid
DPV	differential pulsed voltammetry
*E*	elastic modulus
*E*_p_	peak potential
*F*	Faraday constant
FDM	fused deposition modelling
FFF	fused filament fabrication
GCE	glassy carbon electrode
i3DP	inkjet 3D printing
LOD	limit of detection
LSV	linear sweep voltammetry
MB	Methylene Blue
NH_2_-MWCNT	amino-functionalized multiwalled carbon nanotubes
*R*	universal gas constant
RSD	relative standard deviation
SLA	stereolithography
SPE	screen-printed electrode
SWV	square wave voltammetry
*T*	temperature
TPU	thermoplastic polyurethane
*W*	energy absorption capacity
*ε*_B_	strain at break
*ε*_M_	maximum strain
*σ*_B_	stress at break
*σ*_M_	maximum stress
