# Supplementary material for: A Novel 3D-Printed Flow Cell Design for In Operando Disposable Printed Electrode Replacement: Improving Continuous Methylene Blue Determination
Source: Micromachines (Basel). 2026 Mar 5;17(3):325. doi: 10.3390/mi17030325 (PMC13029348; doi:10.3390/mi17030325)
Supplement: Supplementary file 1 [file micromachines-17-00325-s001.zip › Supplementary information.pdf]

A novel 3D-printed flow cell design for *in operando* disposable printed electrode replacement: improving continuous Methylene Blue determination

Supplementary information

Željka Boček, Elizabeta Forjan, Andrej Molnar, Marijan-Pere Marković, Domagoj Vrsaljko, Petar Kassal*

University of Zagreb, Faculty of Chemical Engineering & Technology, Trg Marka Marulića 19, 10000 Zagreb, Croatia

Correspondence: pkassal@fkit.unizg.hr

Electrochemical method optimization

For all methods except SWV, the primary parameter for optimization was scan rate. For SWV, the primary parameter was frequency, which was ranged from 1000 Hz to 10 Hz, with a constant amplitude of 25 mV.

As shown in Figure S1, CV proved unsuitable due to the capacitive nature of the carbon electrode substrate; at 1 μM MB and at low scan rates, the expected pair of peaks matching the oxidation and reduction of MB ($E_{1/2}$ ~ -0.25 V vs. Ag/AgCl) was completely absent or covered up by the charging current. At higher scan rates, the peaks are visible but poorly defined and low in current magnitude.

LSV showed more promising results. A reduction signal is evident at around -0.2 V vs external Ag/AgCl, especially at higher scan rates (Figure S1B). However, the signal resembles a broad shoulder more than a well-defined peak, and the magnitude of the observed current is very low. Hence, this method was ultimately abandoned as well.

DPV results were inadequate regardless of the parameters applied. Even though all measurements were conducted in a Faraday cage, the noise present in the voltammograms introduced unacceptable irreproducibility at almost all scan rates. Moreover, the peak current magnitude does not improve significantly with scan rate and remains mostly constant (0.3-0.7 μA).

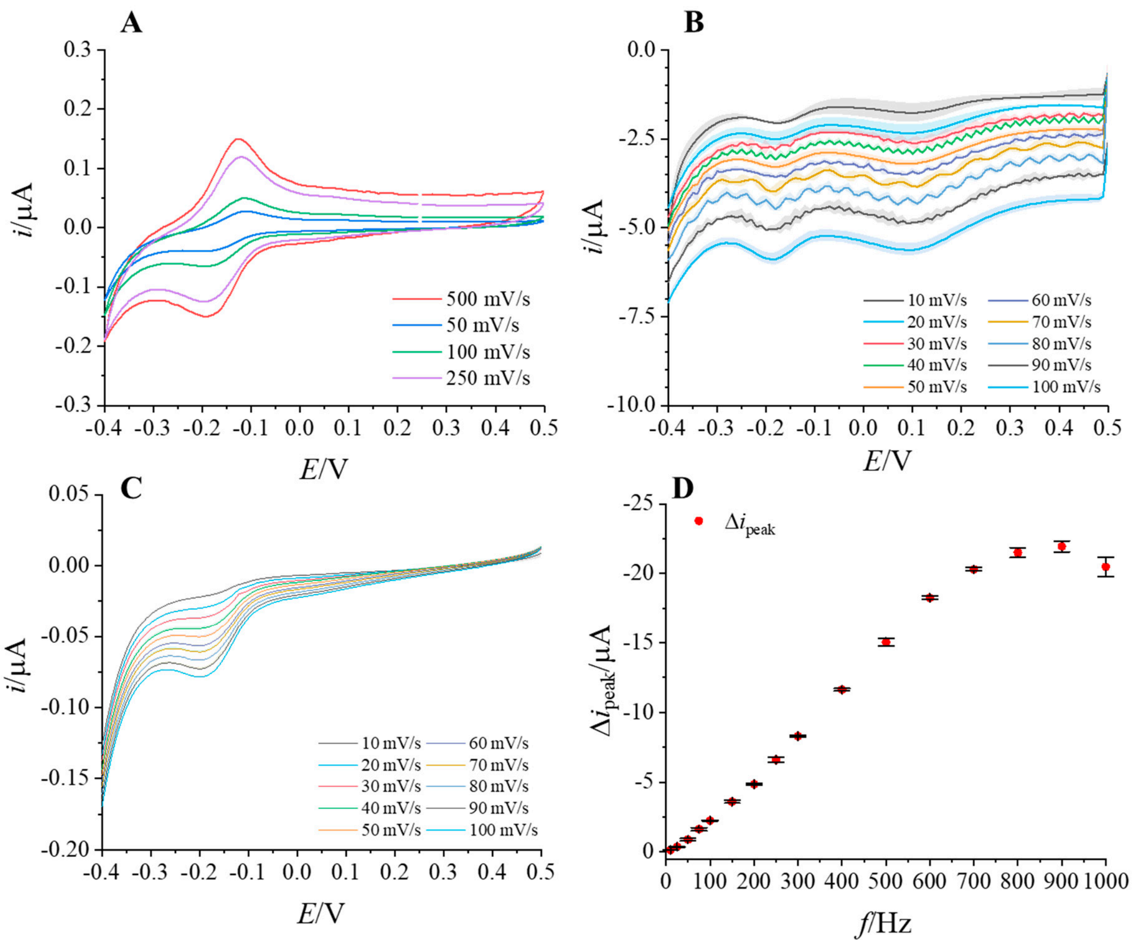

Figure S1. Electrochemical screening results for cyclic voltammetry (A), differential pulsed voltammetry (B) and linear sweep voltammetry (C) conducted in 1 µM MB, phosphate buffer pH = 6, 0.1 M KCl in potential range from -0.4 to +0.5 V vs Ag/AgCl/3M KCl/3M KCl. (D) Δi_{peak} for each frequency tested in square wave voltammetry screening. Cyclic voltammetry parameters: scan rate variable (50, 100, 250 and 500 mV/s), E_{step} 0.01 V, 10 cycles. Differential pulsed voltammetry parameters: scan rate variable (10-100 mV/s), E_{pulse} 0.05 V, t_{pulse} 0.05 s, , E_{step} 0.01 V, E_{begin} +0.5 V, E_{end} -0.4 V. Linear sweep voltammetry parameters: scan rate variable (10-100 mV/s), E_{begin} +0.5 V, E_{end} -0.4 V. Square wave voltammetry parameters: frequency variable (1000-10 Hz), amplitude 0.025 V, E_{step} 0.01 V, E_{begin} +0.5 V, E_{end} -0.4 V.

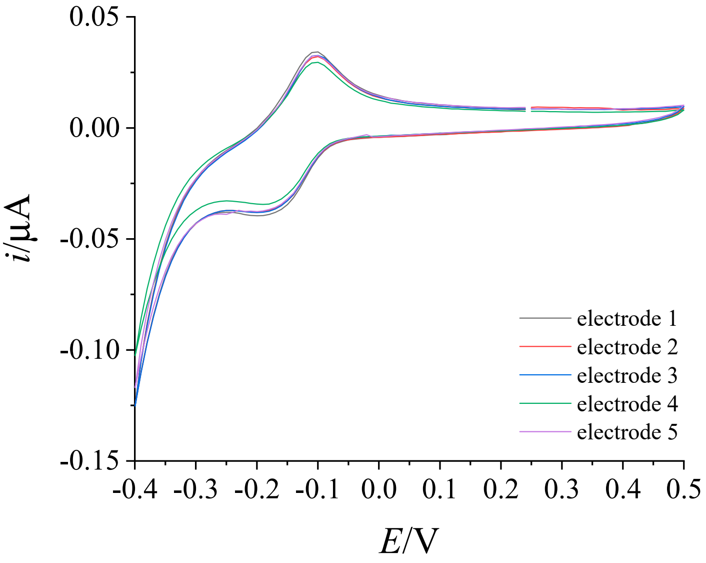

Figure S2. Interelectrode CV reproducibility (*n* = 5) in 1 µM MB, phosphate buffer pH = 6, 0.1 M KCl. Cyclic voltammetry parameters: scan rate 50 mV/s, 10 cycles, potential range from -0.4 to +0.5 V vs. Ag/AgCl/3M KCl/3M KCl.

Preconcentration

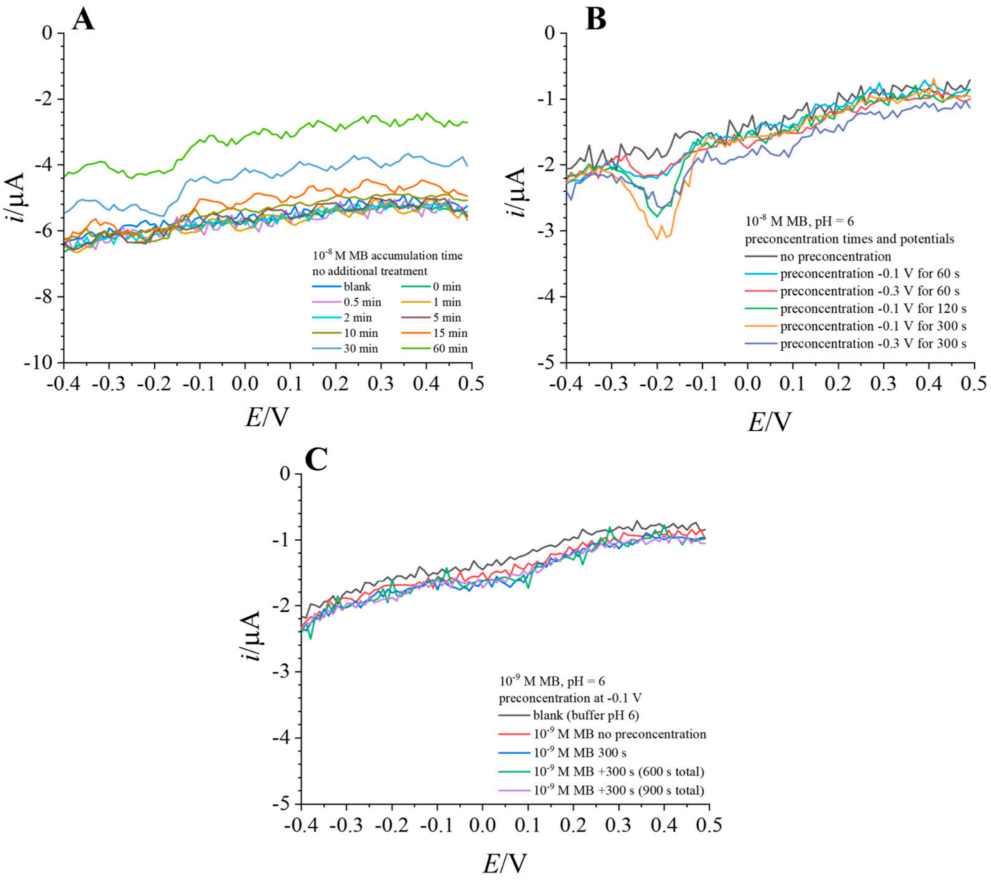

Figure S3. A – Effect of accumulation time in 10^{-8} M MB solution with no additional polarization; B – Effect of different preconcentration times and potentials applied to the working electrode immersed in 10^{-8} M MB solution; C – Effect of preconcentration at -0.1 V applied to the working electrode immersed in 10^{-9} M MB solution.

Intraelectrode reproducibility

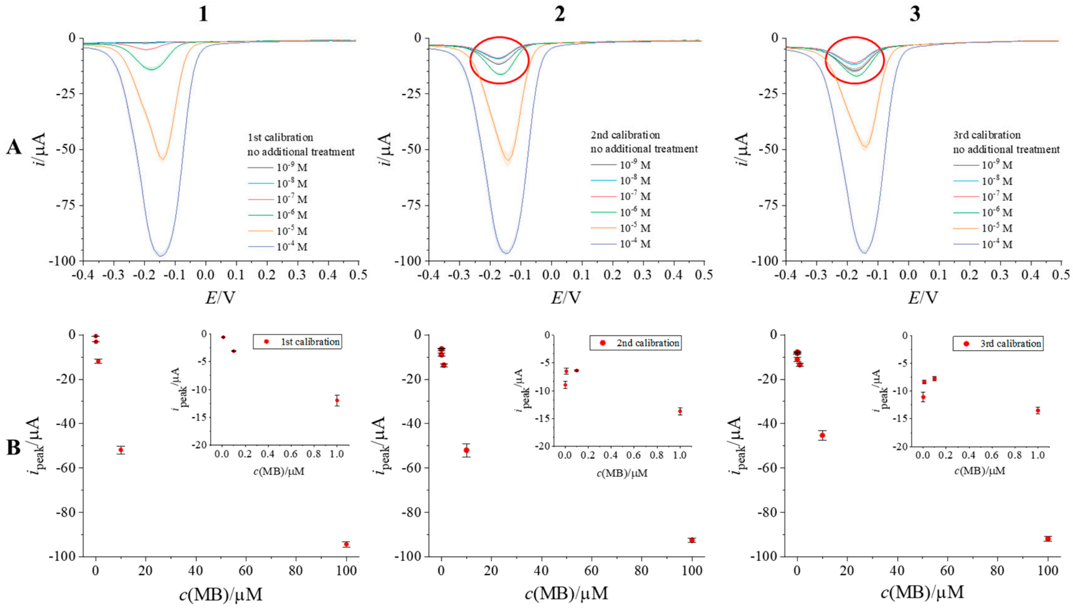

Figure S4. Comparison of three consecutive calibrations conducted with square wave voltammetry (*f* = 600 Hz, pH = 6). Upper row shows the MB reduction cathodic response without any additional treatment applied to the electrode (before or after measurement), while the lower row shows corresponding peak current value dependence on concentration for each voltammogram.

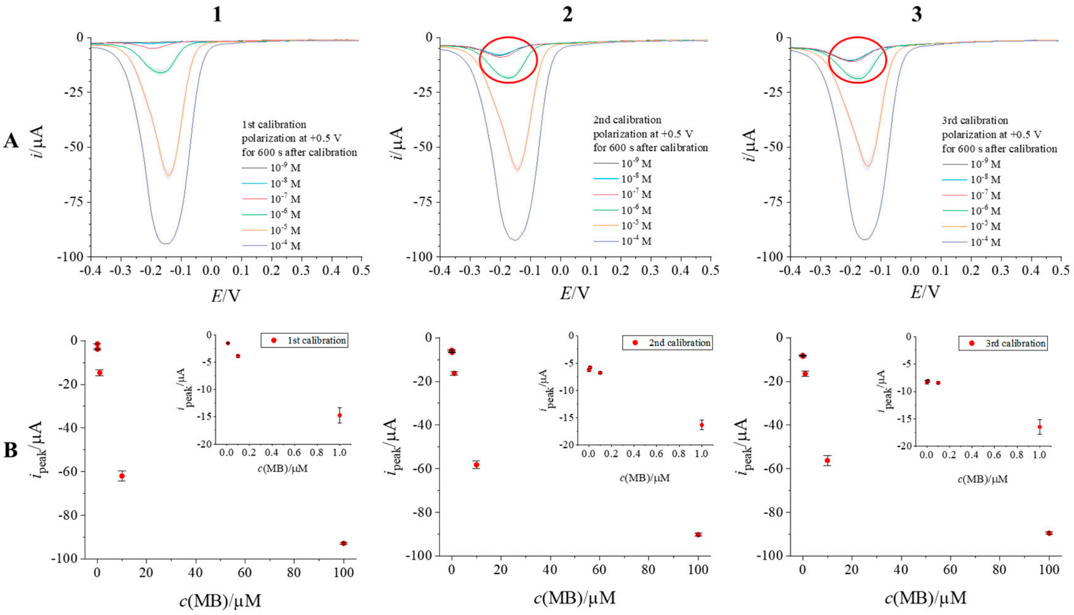

Figure S5. Comparison of three consecutive calibrations conducted with square wave voltammetry (*f* = 600 Hz, pH = 6) with polarization at +0.5 V applied for 600 s in between calibration procedure. Upper row shows the MB reduction cathodic response, while the lower row shows corresponding peak current value dependence on concentration for each voltammogram.

Standard addition method

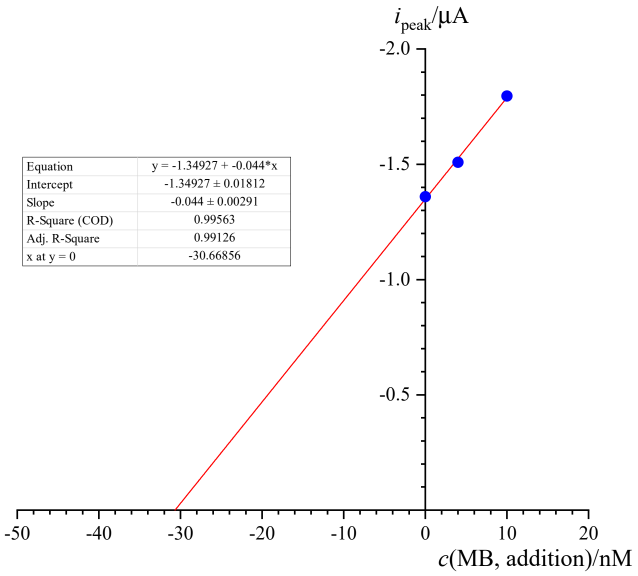

Figure S6. Standard addition method with spiked tap water sample. The recovery through this method was calculated to be 102.23%.

Fourier transform infrared spectroscopy (FTIR)

The FTIR spectrum of the Anycubic Standard Clear resin (Figure S4), predominantly polyacrylate, showed the characteristic absorption bands of this material. A broad band between 3200 cm^{-1} and 3600 cm^{-1} was observed, corresponding to hydroxyl (O–H) stretching vibrations. A strong absorption between 1690 cm^{-1} and 1715 cm^{-1} was also present and is attributed to carbonyl (C=O) stretching vibrations of carboxylic or ketone groups within acrylate phase. An additional band appeared near 1450 cm^{-1}, which corresponds to C–H bending vibrations of methyl groups. A prominent absorption around 1200 cm^{-1} indicates C–O stretching, while another band near 1170 cm^{-1} corresponds to C–O stretching vibrations of aliphatic ether groups from the acrylate. Together, these spectral features confirm the polyacrylate-based chemical composition of the resin [1]. Both uncured and cured resin samples showed identical spectra (Figure S5).

The FTIR spectrum of the thermoplastic polyurethane revealed characteristic functional groups consistent with its chemical structure (Figure S4). A broad absorption band between 3200 cm^{-1} and 3600 cm^{-1} was observed, corresponding to O–H stretching vibrations. In the region from 2800 cm^{-1} to 3200 cm^{-1}, strong doublet bands were detected, characteristic of C–H stretching vibrations in alkyl chains, such as those found in polyalcohols, confirming the aliphatic content of the material. Two additional prominent absorbance bands were identified in the lower wavenumber region. The first, located at approximately 1530 cm^{-1}, corresponds to aromatic nitro group vibrations, while the second, between 1690 cm^{-1} and 1715 cm^{-1}, is associated with carbonyl (C=O) stretching of carboxylic acids or ketone groups. These spectral features are consistent with characteristic structure of TPU and confirm the presence of both aromatic and carbonyl functionalities [2].

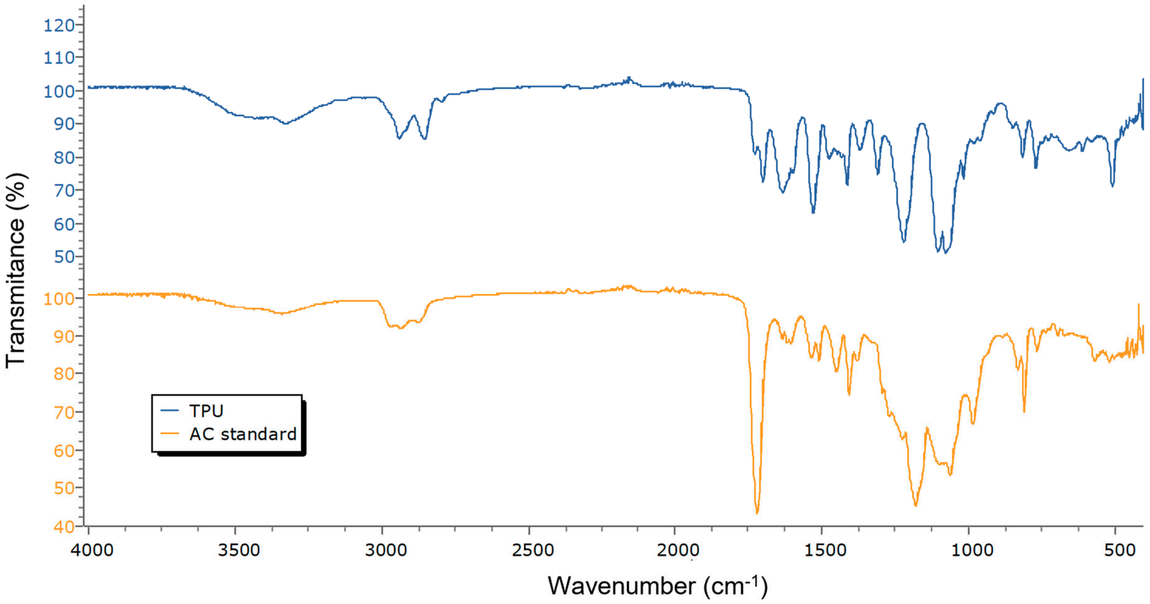

Figure S7. FTIR spectra of Anycubic Standard resin and TPU.

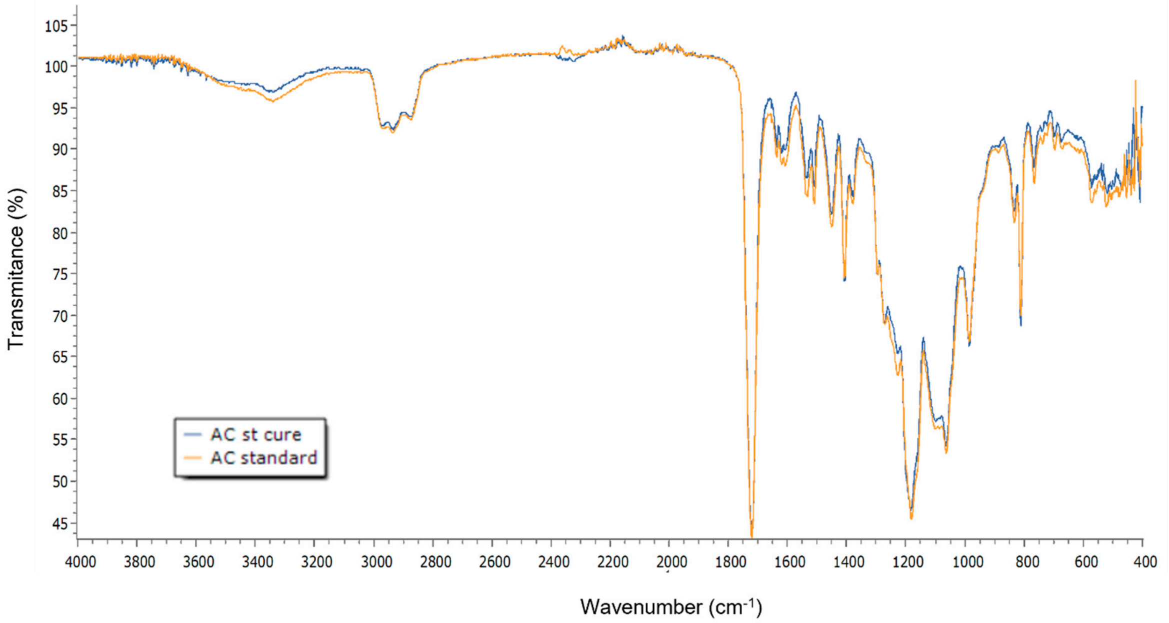

Figure S8. FTIR spectrum of cured Anycubic resin.

REFERENCES

1. B. Smith, *Spectroscopy*, 2023, **38**, 10–14.
2. S. S. Nordi, E. E. M. Noor, C. K. Kok, N. M. Julkapli and M. F. Baig, *Polymers*, 2025, **17**, 899.
